# A New Sesquiterpenoid Hydroquinone from the Marine Sponge *Dysidea arenaria*

**DOI:** 10.3390/molecules13061275

**Published:** 2008-06-06

**Authors:** Yan Qiu, Xiu Min Wang

**Affiliations:** Department of Pharmacy, School of Medical, Xiamen University, Xiamen 361005, People’s Republic of China; E-mail: wangxm@xmu.edu.cn.

**Keywords:** Sesquiterpenoid hydroquinone, anti-HIV activity, *Dysidea arenaria*

## Abstract

Detailed chemical investigation of the South China sponge *Dysidea arenaria* resulted in the isolation of a new sesquiterpenoid hydroquinone, 19-hydroxy-polyfibrospongol B (**1**), along with five known compounds: polyfibrospongol B (**2**), isosemnonorthoquinone (**3**), ilimaquinone (**4**), smenospongine (**5**) and smenotronic acid (**6**). The structures were determined by extensive spectroscopic analysis. The *in vitro* anti-HIV activity on HIV-1 RT was evaluated. Compounds **3-6** displayed moderate inhibitory activity, with IC_50_ values of 239.7, 16.4, 176.1, and 130.4 µM, respectively, while **1** and **2** were found to be inactive against the same biological target.

## Introduction

Numerous sesquiterpenoid quinones and hydroquinones, a still expanding group of C_15_-C_6_ metabolites, have been isolated from marine sponges belonging to the orders Dictyoceratida and Haplosclerida [1]. Such compounds have shown a variety of potentially interesting bioactivities, such as cytotoxicity [2,3,4], antibacterial [3], insecticidal [5], PLA_2_ inhibitor [6], and anti-HIV properties [7]. In their structures, the sesquiterpenoid moiety frequently has a drimane or a 4,9-friedodrimane skeleton. As for the sesquiterpenoid (hydro)quinones, the two moieties in most case contained three methyls, while examples of 14-oxidized compounds are are few in number. The genus *Dysidea* (Dictyoceratida), abundant on tropical reefs in the Indo-Pacific region, generally contains sesquiterpenoids [8], polychlorinated amino acids [9], and polybrominated diphenyl ethers [10], with a rich diversity in structures. As part of our interest in serching for pharmacologically active natural metabolites from marine organisms, the South China Sea marine sponge *Dysidea arenaria* was collected from the coral reefs near Hainan Island. A chemical examination of this sponge afforded a new sesquiterpenoid hydroquinone, 19-hydroxypolyfibrospongol B (**1**), along with five known compounds. In addition, we evaluated *in vitro* anti-HIV activity of the six compounds on HIV-1 RT. 

**Figure 1:**
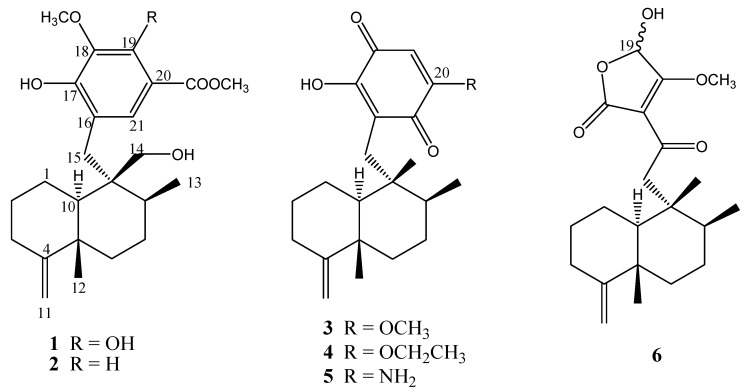
The structures of compounds **1-6**.

## Results and Discussion

The known compounds **2-6** corresponded to polyfibrospongol B (**2**) [11], isosemnonorthoquinone (**3**) [12], ilimaquinone (**4**) [13], smenospongine (**5**) [2], and smenotronic acid (**6**) [14] (Figure 1). The structures of these known sesquiterpenoid (hydro)quinones were identified on the basis of extensive spectroscopic data analysis, and by comparison of their spectral data with those reported in the literature.

**Figure 2:**
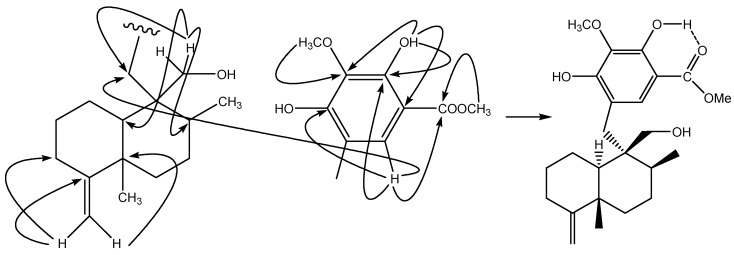
The key HMBC correlations of compound **1**.

19-Hydroxypolyfibrospongol B (**1**), 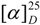 = +2.3 (*c* 0.12, CHCl_3_), was isolated as a white amorphous solid. The molecular formula C_24_H_34_O_6_was deduced on the basis of HRFAB-MS (*m/z*: 417.2277, [M-H]^-^, calcd. 417.2283). The hypothesis of the presence of a phenolic group was confirmed by the IR (3422, 1670, 1440, 1342, 1210, 1056, 985, 892 cm^-1^) and UV (224, 269, 310 nm) data. In its ^1^H-NMR spectrum, the two terminal-vinyl protons at *δ*_H_ 4.44, 4.47 (each 1H, br s), two methyl signals (*δ*_H_ 1.07, s; 1.11, d, *J* = 6.7 Hz), and an additional oxygenated methylene at *δ*_H_ 3.81, 3.90 (d, *J* = 11.7 Hz) suggested the presence of a hydroxymethyl group on a 4,9-friedodrimane-4-ene skeleton. HMBC correlations between the two protons and the carbons at *δ*_C_ 31.0, 37.1, 49.2 located the –CH_2_OH group at C-9. A comparison with the related known compound polyfibrospongol B (**2**) showed a molecular weight 16 a.m.u. greater, which together with the presence of only one aromatic proton at *δ*_H_ 7.38 (1H, s) with a singlet (*δ*_H_ 10.90, 1H, s) at lower fields indicated an additional hydroxyl substitution on the aromatic ring. HMBC correlations: 10.90/105.3, 133.7, 153.0; 7.38/133.7, 153.0, 154.2, 170.7; 3.99/133.7; 3.92/170.7 (Figure 2) confirmed the structure of a pentasubstituted phenolic group. It became clear that the 4,9-friedodrimane skeleton was connected to the aromatic group when the correlation between *δ*_H_ 7.38 and *δ*_C_ 31.0 in the HMBC spectrum was observed. The relative configuration was suggested by the NOESY correlations found between Me-12 and Me-13, and between Me-12 and H-14. The proton at *δ*_H_ 1.06 (1H, m, H-10) was overlapped with Me-12 and Me-13. Detailed elucidation with NOESY spectrum revealed the crosspeak between H-10 and H-15a (1H, d, *J* = 15 Hz), while no crosspeaks were observed between H-10 and H_2_-14, which indicated the same orientation of H-10 and H_2_-15.

**Table 1:** ^13^C-NMR (125 MHz) and ^1^H-NMR (500 MHz) data of Compound **1** (CDCl_3_) .

No.	Compound **1**		No.	Compound **1**	
	C	H (*J* Hz)		C	H (*J* Hz)
1	24.1	2.12, m	13	18.9	1.11, d, (6.7)
2	28.3	1.85, m	14	64.5	3.90, d, (11.7) 3.81, d, (11.7)
3	33.2	2.33, m 2.12, m	15	31.0	3.02, d, (15.0) 2.77, d, (15.0)
4	159.7		16	116.4	
5	40.0		17	154.2	
6	36.9	1.60, m	18	133.7	
7	27.9	1.47, m	19	153.0	
8	37.1	1.30, m	20	105.3	
9	46.2		21	128.6	7.38, s
10	49.2	1.06, m	18-OMe	60.8	3.99, s
11	103.3	4.47, 4.44, br s	20-COOMe	52.1	3.92, s
12	20.9	1.08, s		170.7	

Anti-HIV activities of compounds **1-6** were evaluated by their inhibition of HIV-1 RT. As the positive control, PFA expressed the inhibition ratio of 85% at 0.05 µM. Compounds **3-6** displayed very weak inhibitions, with IC_50_ values of 239.7, 16.4, 176.1, and 130.4 µM, respectively, while compounds **1** and **2** were inactive against the same biological target. The different activity profiles observed for above compounds suggested that some of the structural elements present might be responsible for HIV-1 RT inhibition, and our structure-activity relationship analysis indicated that the existence of a quinone ring might be important for expression of the anti-HIV activity. When the quinone ring was reduced to a hydroquinone ring, no bioactivity could be detected at a concentration of 200 µg/mL.

## Experimental

### General

The IR spectra were determined on a Thermo Nicolet Nexus 470 FT-IR spectrometer. Optical rotations were measured with a Perkin-Elmer 243 B polarimeter using a 1 dm microcell. The ^1^H-NMR and ^13^C-NMR spectra were recorded on a Bruker Avance-500 FT NMR spectrometer. HRFAB-MS spectra were performed with a Bruker APEX II mass spectrometer. ESI-MS were recorded on a PE Q-STAR ESI-TOF-MS/MS spectrometer. Column chromatography was carried with silica gel (200-300 mesh), and HF_254_ silica gel for TLC was obtained from Qingdao Marine Chemistry Co. Ltd. (Qingdao, People’s Republic of China). ODS and Sephadex LH-20 (18-110 μm) was obtained from Pharmacia Co.

### Extraction and Isolation

The specimen of *Dysidea* *arenaria* was collected from Hainan Island, South China Sea, China, in January 2006. A voucher specimen (MSB-7) is deposited at the Department of Pharmacy, School of Medical, Xiamen University. The sponge (216 g, dry wt.), which had been immersed in EtOH, was homogenized and then extracted with MeOH. The concentrated total extract (7.7 g) was partitioned between CHCl_3_ and H_2_O, and then the CHCl_3_-soluble portion (4.6 g) was repartitioned between petroleum ether and 90% MeOH. The 90% MeOH extract (2.33 g) was subjected to flash silica gel column chromatography eluted with a petroleum ether-ethyl acetate stepwise gradient to give three fractions. Fraction 2 (1.11 g) was applied to a silica gel column eluted with petroleum ether–acetone (3:1) to give five fractions. Fr. 2-1 was subjected to ODS column (85% MeOH) and then Sephadex LH-20 chromatography eluted with MeOH to give crude needles, which were repurified on a silica gel column (petroleum ether-ethyl acetate 3:1) to give compound **3** (5.6 mg). With the same procedures above, **4** (54 mg) was obtained from Fr. 2-2. Fr. 2-3 was filtered through a Sephadex LH-20 column with MeOH to produce four fractions identified as Fr. 2-3(a, b, c, d). The last one, Fr. 2-3d, was pure compound **5** (72 mg). Fr. 2-3b and 2-3c were purified by repeated silica gel column chromatography (petroleum ether-ethyl acetate) to give **6** (58 mg), **1** (14 mg), and **2** (6.8 mg). 

*Compound* **1**: white amorphous solid, 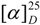 = +2.3 (*c* 0.12, CHCl_3_); UV (MeOH) *λ*_max_ (log *ε*) 224 (4.55), 269 (4.26), 310 (3.78) nm; IR (neat) *ν*_max_ 3422, 1670, 1440, 1342, 1210, 1056, 985, 892, 795 cm^-1^; for ^1^H-NMR and ^13^C-NMR see Table 1; ESI-TOF MS (*m/z*): 417 [M-H]^-^, 403 [M-Me]^-^, 386; HRFAB-MS (*m/z*) 417.2277, [M-H]^-^, (calcd. for C_24_H_3__3_O_6_, 417.2283).

*Compound* **2**: white amorphous solid, 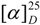 = +1.7 (*c* 0.26, CHCl_3_); UV (MeOH) λmax (log *ε*) 227 (4.57), 270 (4.21), 301 (3.82) nm; IR (neat) ν_max_ 3420, 2940, 1710, 1645, 1440, 1310, 1220, 1025 cm^-1^; ESI-TOF MS^-^ (m/z): 401 [M-H]^-^, 386[M-H-Me]^-^; ^1^H-NMR (CDCl_3_): *δ*_H_ 2.15 (2H, dd, *J* = 13.5, 2.0 Hz, H-1), 1.90, 1.35 (each 1H, m, H-2), 2.30, 2.10 (each 1H, m, H-3), 1.60, 1.57 (each 1H, m, H-6), 1.48 (2H, m, H-7), 1.40 (1H, m, H-8), 1.12 (1H, m, H-10), 4.46, 4.42 (each 1H, br s, H-11), 1.08 (3H, s, H-12), 1.13 (1H, d, *J* = 6.5 Hz, H-13), 3.92, 3.82 (each 1H, d, *J* = 11.7 Hz, H-14), 3.14, 2.86 (each 1H, d, *J =*14.5 Hz, H-15), 7.42 (1H, d, *J* = 1.7 Hz, H-19), 7.52 (1H, d, *J* = 1.7 Hz, H-21), 3.96 (3H, s, 18-OMe), 3.90 (3H, s, 20-COOMe).

*Compound* **3**: orange needles, 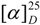 = -23.2 (*c* 1.23, CHCl_3_); UV (MeOH) λ_max_ (log *ε*) 285 (4.36), 420 (3.12) nm; IR (neat) ν_max_ 3340, 1642, 1607, 1205 cm^-1^; ESI-TOF MS^-^ (m/z): 357 [M-H]^-^; ^1^H-NMR (CDCl_3_): *δ*_H_ 2.10, 1.44 (each 1H, m, H-1), 1.86, 1.18 (each 1H, m, H-2), 2.32, 2.08 (each 1H, ddd, *J* = 13.7, 8.6, 5.4 Hz, H-3), 1.51, 1.34 (each 1H, m, H-6), 1.39 (2H, m, H-7), 1.16 (1H, m, H-8), 0.76 (1H, dd, *J* = 12.0, 2.0 Hz, H-10), 4.33, 4.34 (each 1H, br s, H-11), 1.04 (3H, s, H-12), 0.98 (3H, d, *J* = 6.5 Hz, H-13), 0.84 (3H, s, H-14), 2.53, 2.47 (each 1H, d, *J* = 13.7 Hz, H-15), 5.86 (1H, s, H-19), 3.86 (3H, s, 20-OMe).

*Compound* **4**: orange needles, 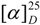 = -20.4 (*c* 0.82, CHCl_3_); UV (MeOH) λ_max_ (log *ε*) 277 (4.38), 422 (3.17) nm; IR (neat) ν_max_ 3338, 2924, 2856, 1645, 1609, 1382, 1234, 1220 cm^-1^; HRFAB-MS^-^ (m/z): 371.2224 [M-1]^-^; ^1^H-NMR (CDCl_3_): *δ*_H_ 2.10, 1.47 (each 1H, m, H-1), 1.88, 1.19 (each 1H, m, H-2), 2.35, 2.09 (each 1H, ddd, *J* = 13.8, 8.5, 5.5 Hz, H-3), 1.55, 1.36 (each 1H, m, H-6), 1.41 (2H, m, H-7), 1.17 (1H, m, H-8), 0.80 (1H, dd, *J* = 12.0, 1.7 Hz, H-10), 4.46, 4.47 (each 1H, br s, H-11), 1.07 (3H, s, H-12), 1.00 (3H, d, *J* = 6.0 Hz, H-13), 0.87 (3H, s, H-14), 2.54, 2.49 (each 1H, d, *J* = 13.5 Hz, H-15), 5.85 (1H, s, H-19), 4.07 (2H, q, *J* = 7.0 Hz, 20-OCH_2_CH_3_), 1.52 (3H, t, *J* = 7.0 Hz, 20-OCH_2_CH_3_).

*Compound* **5**: purple needles, 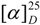 = -17.6 (*c* 0.82, MeOH); UV (MeOH) λ_max_ (log *ε*) 206 (4.14), 317 (3.52) nm; IR (neat) ν_max_ 3477, 3280, 2921, 2858, 1568, 1375, 1333, 1203 cm^-1^; ESI-TOF MS^-^ (m/z): 342 [M-H]^-^; ^1^H-NMR (CDCl_3_): *δ*_H_ 2.05, 1.47 (each 1H, m, H-1), 1.89, 1.26 (each 1H, m, H-2), 2.53, 2.34 (each 1H, m, H-3), 1.56, 1.32 (each 1H, m, H-6), 1.40 (2H, m, H-7), 1.17 (1H, m, H-8), 1.33 (1H, m, H-10), 4.47 (2H, br s, H-11), 1.06 (3H, s, H-12), 1.23 (3H, d, *J* = 6.3 Hz, H-13), 0.94 (3H, s, H-14), 2.81, 2.72 (each 1H, d, *J* = 13.6 Hz, H-15), 5.86 (1H, s, H-19).

*Compound* **6**: a white amorphous, solid, was isolated as a 1:1 mixture of the two 19-epimers. UV (MeOH) λ_max_ (log *ε*) 224 (4.54) nm; IR (neat) ν_max_ 3414, 1795, 1770, 1722 cm^-1^; ESI-TOF MS^-^ (m/z): 361[M-H]^-^; ^1^H-NMR (CDCl_3_): *δ*_H_ 1.44, 1.22 (each 1H, m, H-1), 1.84, 1.37 (each 1H, m, H-2), 2.26, 2.10 (each 1H, m, H-3), 1.60 (2H, m, H-6), 1.47 (2H, m, H-7), 2.01 (1H, m, H-8), 1.66 (1H, dd, *J* = 12.4, 2.3 Hz, H-10), 4.51 (2H, br s, H-11), 1.02 (3H, s, H-12), 0.77 (3H, d, *J* = 6.0 Hz, H-13), 0.76 (3H, s, H-14), 2.63, 2.51 (each 1H, d, *J* = 19.0 Hz, H-15), 5.30 (1H, s, H-19), 3.92 (3H, s, H-21); ^1^H-NMR of the 19-epimer (CDCl_3_): *δ*_H_ 1.44, 1.22 (each 1H, m, H-1), 1.84, 1.37 (each 1H, m, H-2), 2.26, 2.10 (each 1H, m, H-3), 1.60 (2H, m, H-6), 1.47 (2H, m, H-7), 2.01 (1H, m, H-8), 1.72 (1H, dd, *J* = 12.4, 2.3 Hz, H-10), 4.53, 4.51 (each 1H, br s, H-11), 1.02 (3H, s, H-12), 0.81 (3H, d, *J* = 6.0 Hz, H-13), 0.76 (3H, s, H-14), 2.78, 2.34 (each 1H, d, *J* = 19.0 Hz, H-15), 5.30 (1H, s, H-19), 3.93 (3H, s, H-21).
